# G3BP1 interacts with YWHAZ to regulate chemoresistance and predict adjuvant chemotherapy benefit in gastric cancer

**DOI:** 10.1038/s41416-020-01067-1

**Published:** 2020-09-29

**Authors:** Junjie Zhao, Xuhong Fu, Hao Chen, Lingqiang Min, Jie Sun, Jingyi Yin, Jianping Guo, Haojie Li, Zhaoqing Tang, Yuanyuan Ruan, Xuefei Wang, Yihong Sun, Liyu Huang

**Affiliations:** 1 Department of General Surgery, Zhongshan Hospital, Fudan University, 200032 Shanghai, China; 2 Cancer Center, Zhongshan Hospital, Fudan University, 200032 Shanghai, China; 3 Gastric cancer center, Zhongshan Hospital, Fudan University, 200032 Shanghai, China; 4 Key Laboratory of Systems Biomedicine (Ministry of Education) and Collaborative Innovation Center of Systems Biomedicine, Shanghai Center for Systems Biomedicine, Shanghai Jiao Tong University, 200240 Shanghai, China; 5 Institute of Precision Medicine, the First Affiliated Hospital, Sun Yat-sen University, Guangzhou, Guangdong 510275 China; 6 Department of Biochemistry and Molecular Biology, School of Basic Medical Sciences, Fudan University, 200032 Shanghai, China

**Keywords:** Gastric cancer, Cell death

## Abstract

**BACKGROUND:**

A large proportion of gastric cancer patients are susceptible to chemoresistance, while the underlying mechanism remains obscure. Stress granules (SGs) play a self-defence role for tumour cells in inhibiting chemotherapy-induced apoptosis. As an SG assembly effector, G3BP1 (Ras-GTPase-activating protein SH3 domain-binding protein) has been reported to be overexpressed in gastric cancer; thus, here we aim to explore its potent roles in gastric cancer chemoresistance.

**METHODS:**

Kaplan–Meier analysis was used to compare survival rates in gastric cancer patients with different G3BP1 expression. The influence of G3BP1 on gastric cancer cell chemoresistance and apoptosis were evaluated by in vitro and in vivo approaches. The interaction between G3BP1 and YWHAZ was assessed by immunohistochemistry, immunoprecipitation and immunofluorescence.

**RESULTS:**

G3BP1 was associated with the poor outcome of gastric cancer patients who received adjuvant chemotherapy. *G3BP1* knockdown significantly increased the sensitivity of gastric cancer cells to chemotherapy drugs. Mechanically, cell apoptosis and pro-apoptotic-associated molecules were significantly elevated upon *G3BP1* depletion. Gene co-expression network analyses identified YWHAZ as the critical interlayer of G3BP1; as a result, G3BP1 interacted with YWHAZ to sequester Bax into the cytoplasm. Clinically, G3BP1^high^YWHAZ^high^ gastric cancer patients displayed the worst outcome compared with other patients after chemotherapy.

**CONCLUSIONS:**

The expression of G3BP1 and YWHAZ could predict the adjuvant chemotherapy benefit in gastric cancer patients.

## BACKGROUND

Most patients with gastric cancer in China and Western countries are asymptomatic and already have a disease that extends beyond locoregional confines when primarily diagnosed.^1^ Despite the availability of multiple disciplinary treatments, including surgery, chemotherapy, radiotherapy and immunotherapy, the 5-year survival rate for patients with advanced gastric cancer remains poor due to the high possibility of recurrence and metastasis.^2–4^ Based on accumulating evidence, the standard clinical treatments are potentially curative surgery with conventional adjuvant or neoadjuvant chemotherapy for resectable advanced gastric cancer,^5–8^ or palliative chemotherapy for unresectable gastric cancer.^9^ However, the large patient cohorts involved in the randomised controlled trials (RCTs) are heterogeneous populations that may lead to difficulties to interpret for every specific subgroup.^10^ Therefore, even in the previous RCTs that demonstrated a survival benefit with chemotherapy, capecitabine and oxaliplatin could only reduce the hazard ratio of 5-year disease-free survival to 0.58,^7^ and the overall response rates were only 40% for SOX (S-1 plus oxaliplatin), and 44% for CAPOX (capecitabine plus oxaliplatin) in the advanced gastric cancer.^11^ A large proportion of patients are susceptible to chemoresistance, resulting in tumour recurrence and metastasis after adjuvant chemotherapy (ACT).^12^

Upon exposure to environmental stress such as chemotherapy, cells generally invoke a defence mechanism to inhibit apoptosis and in turn to repair the stress-induced alterations via stress granules (SGs) formation.^13^ G3BP1 (Ras-GTPase-activating protein SH3 domain-binding protein) has been demonstrated to function as an SGs assembly effector, regulating mRNA turnover and determining the fate of mRNAs during cellular stress.^14,15^ In addition, G3BP1 was also reported to bind the SH3 domain of Ras-GAP and participate in the regulation of the *RAS* signalling pathway.^16^ Recently, G3BP1 was found to promote cytosolic DNA binding and activation of cyclic GMP-AMP synthase (cGAS), which is critical for cGAS-mediated interferon production.^17^ More importantly, G3BP1 has been demonstrated to play important oncogenic roles in tumorigenesis, and is highly expressed in multiple tumours, including liver, breast and colon cancers.^18^ For example, G3BP1 could negatively regulate p53 tumour suppressor roles in tumorigenesis, either by interacting with p53-deubiquitinating enzyme ubiquitin-specific protease 10 to inhibit melanoma cell apoptosis^18,19^ or by interacting with long non-coding RNA P53RRA to enhance p53 nuclear retention to promote lung cancer ferroptosis and apoptosis.^20^

In our study, we found that high G3BP1 expression was markedly correlated with poor survival of gastric cancer patients who received ACT and revealed a potent oncogenic role for G3BP1 in apoptosis inhibition and chemoresistance in gastric cancer.

## METHODS

### Patients and follow-up

Four hundred and fifty-five gastric cancer patients who had undergone surgery in Zhongshan Hospital, Fudan University between 2004 and 2008 were enrolled in the study. Clinicopathological parameters of each patient, including age, gender, tumour size, location, tumour differentiation, Lauren classification, vessel invasion and TNM stage, were retrospectively collected from the medical record. Overall survival (OS) was defined as the interval between the date of surgery and death or the last visit. All the patients were followed up until April 2014 with the median follow-up time of 41 months. The study was approved by the Research Ethics Committee of Fudan University and was performed in accordance with the ethical standards laid down in the 1964 Declaration of Helsinki and its later amendments. Written informed consents from each patient were achieved.

### Tissue microarrays and immunohistochemistry (IHC)

Tissue microarray construction and immunohistochemical staining were performed as previously described.^21^ Primary anti-G3BP1 (Santa Cruz, sc-81940, 1:100 dilution) and anti-YWHAZ (Santa Cruz, sc-1019, 1:50 dilution) antibodies were used for IHC staining. The stained sections were assessed by two independent pathologists who were blind to patients’ clinical data. The staining extent was categorised as follows: 0, <5%; 1, 5–25%; 2, 26–50%; 3, 51–75%; 4, >75%. The intensity was categorised as follows: 0, negative; 1, weak; 2, moderate; 3, strong. The IHC staining score (a series of results ranging from 0 to 12) was calculated by multiplying staining extent and staining intensity. According to receiver operating characteristic analysis, a score >4 was considered as a high expression for G3BP1 and YWHAZ.

### Cells, antibodies and reagents

The human gastric cancer cell lines MGC80-3, HGC-27, SGC-7901 and BGC-823 were purchased from the Cell Bank of the Type Culture Collection of the Chinese Academy of Sciences (Shanghai, China), and were cultured in Dulbecco’s modified Eagle’s medium or RPMI-1640 supplemented with 10% foetal bovine serum (catalogue no. 16000-044; Gibco, Grand Island, NY, USA) at 37 °C in a humidified atmosphere containing 5% CO_2_. The primary antibodies including cleaved PARP (5625S), cleaved caspase-9 (9501S), cleaved caspase-3 (9664S) and Bax (2772) were purchased from Cell Signalling Technology (Beverly, MA, USA); G3BP1 (sc-81940) and YWHAZ (sc-1019) were purchased from Santa Cruz (Dallas, TX, USA); Bax (60267-1-Ig, 50599-2-Ig), eIF4D (10219-1-AP) and HA (51064-2AP) were purchased from Proteintech (Wuhan, Hubei, China); COX IV (GTX628901) were purchased from GeneTex (Irvine, CA, USA). The chemotherapeutic reagents, capecitabine and oxaliplatin, were purchased from Meilun Biotech Co., Ltd (Dalian, China); Epigallocatechin gallate (EGCG) and actinomysin D were purchased from MedChemExpress (Monmouth Junction, NJ, USA).

### RNA interference, plasmids and transfection

The small interfering RNA (siRNA) (si*G3BP1*-1: 5′-GCGCAUUAACAGUGGUGGGAAAUUA-3′; si*G3BP1*-2: 5′-ACAUUUAGAGGAGCCUGUUGCUGAA-3′; si*YWHAZ*: 5′-GGAGAUUACUACCGUUACU-3′; si*Bax*: 5′-AAGGUGCCGGAACUGAUCAGA-3′) and negative control siRNA (5′UUACGGAUCGACUUAAGCCGUUGCA-3′) were constructed by Biotend Research (Shanghai, China). The short hairpin RNA sequence of pLKO vectors to deplete *G3BP1* is: sense, 5′-GCGCATTAACAGTGGTGGGAAATTA-3′. The pcDNA3-G3BP1 plasmid was constructed by Youbio Company (Changsha, Hunan, China). Different fragments of G3BP1 were generated by subcloning the corresponding cDNAs into the pcDNA3-HA-vector. pCDH-GFP-G3BP1 were constructed by cloning the full-length G3BP1 cDNA into the pCDH vector. Transfections were performed with Lipofectamine 3000 (Life Technologies, Carlsbad, CA, USA) according to the manufacturer’s instructions.

### Western blotting

Cell lysates were separated by sodium dodecyl sulfate-polyacrylamide gel electrophoresis and transferred onto polyvinylidene difluoride membranes. After that, membranes were incubated with primary antibodies and followed by horseradish peroxidase-conjugated secondary antibody. Protein expression was detected by enhanced chemiluminescence assay (GE Amersham Imager 600).

### Mitochondria isolation

Gastric cancer cells MGC80-3 were transfected with scramble or si*G3BP1*. After culturing for 48 h in 100 mm dishes, cells were collected and washed with phosphate-buffered saline (PBS). Then, cells were lysed in 100 μL Permeabilisation buffer (20 mM HEPES pH 7.2, 100 mM KCl, 5 mM MgCl_2_, 1 mM EGTA, 250 mM sucrose, 0.01% digitonin) and incubated at 4 °C for 15 min. After centrifugation (16,000 × *g* for 10 min), supernatants were removed from each sample as “cytoplasm lysis” and cell pellets were resuspended in 100 μL of ice-cold ONYX buffer (20 mM Tris pH 7.4, 135 mM NaCl, 1.5 mM MgCl_2_, 1 mM EGTA, 10% v/v glycerol, 1% Triton X-100, protease inhibitor). Supernatants were collected as “mitochondria lysis” by centrifugation at 16,000 × *g* for 10 min. The lysates were boiled with 4× SDS laemmli buffer for immunoblotting analysis.

### Cytotoxicity assay

Cell cytotoxicity was determined by Cell Counting Kit-8 (CCK-8) assay (Beyotime, Nantong, China). Briefly, gastric cancer cells were transfected as indicated. After 48 h, transfected cells were seeded in 96-well plates at 5 × 10^4^ cells/well and incubated with chemotherapeutic agents (capecitabine: 32–1024 µg/mL; oxaliplatin: 8–256 µg/mL). After an indicated time of incubation, 10 μL CCK-8 was added in the medium for 1 h. Absorbance was determined at 450 nm with a Universal Microplate Reader (Bio-Tek Instruments, Winooski, VT, USA). The relative chemoresistance folds were analysed by compared with IC_50_ (half-maximal inhibitory concentration) values. All assays were performed in triplicate.

### Cell apoptosis and cell cycle assay

Cell apoptosis and cell cycle ratios were detected by flow cytometric analysis. After transfection for 48 h and incubation with capecitabine (64 µg/mL) or oxaliplatin (8 µg/mL) for 12 h, cells were digested with trypsin and washed twice with ice-cold PBS. Cellular apoptosis was determined by FITC Annexin V and propidium iodide (PI) using FITC Annexin V Apoptosis Detection Kit I (BD Biosciences, CA, USA). In cell cycle assay, cells were stained with PI (P-4170, Sigma-Aldrich, Germany) and were assessed by flow cytometry. The data were analysed by the FlowJo software (TreeStar, Ashland, OR, USA). All assays were performed in triplicate.

### Colony formation assay

Cells were plated in 6-well dishes at a density of 500 cells per well and were treated with capecitabine (16 µg/mL) or oxaliplatin (2 µg/mL). Two weeks later, cells were fixed and stained with crystal violet on the plates.

### Confocal microscopy

Pre-treated gastric cancer cells were seeded on glass coverslips overnight. Then, cells were washed twice with PBS, fixed with 4% paraformaldehyde for 20 min and permeabilised with 0.1% Triton X-100 for 8 min at room temperature. After that, cells were incubated with specific primary antibodies for 2 h at room temperature, followed by incubation with fluorescent secondary antibodies (Jackson). Nuclei were stained with DAPI (4′,6-diamidino-2-phenylindole). The cells were visualised by laser confocal scanning microscope (Leica Microsystems Heidelberg GmbH, Germany).

### Co-immunoprecipitation

Cells were grown to 80–90% density in 100 mm dishes, and then were collected and washed with PBS. All cells were solubilised with IP lysis buffer (50 mM Tris-HCl pH 7.5, 150 mM NaCl, 0.1% NP-40, 15 mM MgCl_2_, 10 μg/mL aprotinin, 10 μg/mL leupeptin, 1 mM phenylmethylsulfonyl fluoride). Whole-cell lysates were incubated with 2 μg relevant antibodies at 4 °C for 2 h. Pre-equilibrated protein G agarose beads were added and collected by centrifugation after incubation overnight and then gently washed three times with the lysis buffer. The bound proteins were eluted and analysed using Western blotting.

### Tumorigenesis in xenograft mouse model

The animal experiments were approved by the Animal Ethics Committee of Zhongshan Hospital and were performed in accordance with the Guidelines for Animal Health and Use. All sections of this report adhere to the ARRIVE Guidelines for reporting animal research. A completed ARRIVE guidelines checklist is included in Supplementary Checklist. Balb/c nude mice and nonobese diabetic/severe-combined immunodeficiency (NOD-SCID) mice were purchased from Shanghai Laboratory Animal Centre of Chinese Academy Sciences and were housed in individually ventilated cages with free access to water and food in a specific pathogen-free room. MGC80-3 cells (1 × 10^7^ cells in 100 μL PBS), which were stably transfected with the indicated plasmids, were injected subcutaneously into the flanks of 6-week-old male Balb/c nude mice. Starting 2 weeks later, mice were randomly allocated into the chemotherapy group (injection with 200 mg/kg capecitabine intraperitoneally, 3 times per week) or control group (normal saline). At 6 weeks post injection, the Balb/c nude mice were sacrificed by cervical dislocation after carbon dioxide inhalation, and tumours were collected and weighted. In a rescue experiment, we used 6-week-old male NOD-SCID mice. At 6 days after the injection of three different groups of MGC80-3 cells (1 × 10^7^), the NOD-SCID mice were injected with capecitabine intraperitoneally (100 mg/kg, 2 times per week). Three weeks later, the mice were, respectively, sacrificed by cervical dislocation after carbon dioxide inhalation, and tumours were harvested.

### Statistical analysis

Statistical analysis was performed with SPSS (version 19, Chicago, IL, USA). Categorical variables were analysed by using Pearson’s test or Fisher’s exact test. Continuous variables were analysed by Student’s *t* test. Two-way analysis of variance was used to compare the difference between groups in animal experiments. Kaplan–Meier analysis was used to determine the survival rate, and the log-rank test was used to compare two survival rate curves. Correlations between G3BP1 and YWHAZ expression was calculated by Spearman’s correlation analysis. All statistical analyses were two-sided, and *P* < 0.05 was considered statistically significant.

## RESULTS

### High G3BP1 correlates with poor survival of gastric cancer patients who received chemotherapy

**Fig. 1:**
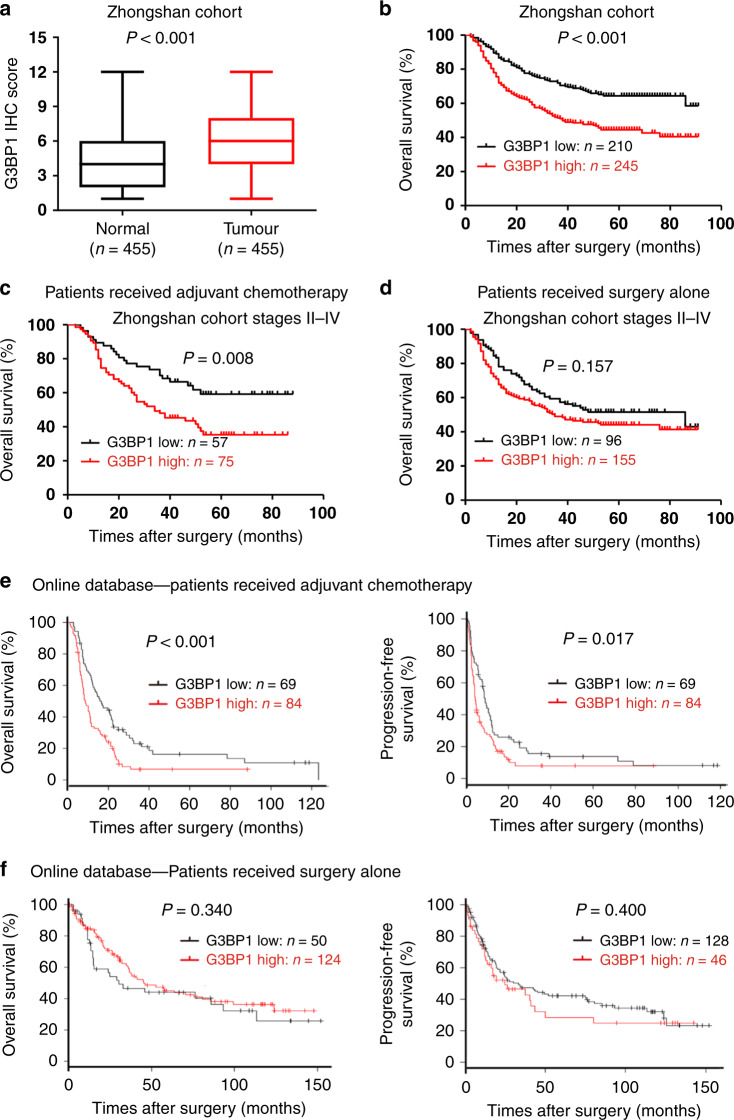
High G3BP1 expression is correlated with poor survival of gastric cancer patients who received adjuvant chemotherapy. **a** G3BP1 IHC scores in gastric cancer and normal tissues (*n* = 455) from the Zhongshan cohort. The box plot showed the full range of variation (error bars: min and max) with the line representing median. **b**–**d** Kaplan–Meier analysis for the overall survival rate of gastric cancer patients from the Zhongshan cohort. **b** The association of G3BP1 expression with the survival of all patients (*n* = 455). **c** The association of G3BP1 expression with the survival of patients receiving adjuvant chemotherapy in stages II–IV (*n* = 132). **d** The association of G3BP1 expression with the survival of patients receiving surgery alone in stages II–IV (*n* = 251). **e**, **f** The association of G3BP1 expression with overall survival and progression-free survival of gastric cancer patients receiving adjuvant chemotherapy (**e**) or surgery alone (**f**) by analysing the integrated online datasets (GSE14210, GSE15459, GSE22377, GSE29272, GSE51105 and GSE62254) (http://www.kmplot.com/analysis/index.php?p=service&cancer=gastric).

By analysing microarray datasets from the Gene Expression Omnibus (GEO) and the Cancer Genome Atlas (TCGA) database, we found that the mRNA level of *G3BP1*, but not that of family member *G3BP2*, was significantly higher in human gastric cancer samples than in normal tissue samples (Supplementary Fig. 1a–e). We then analysed the G3BP1 expression level in gastric cancer samples (*n* = 455) from Zhongshan Hospital. The IHC results showed that G3BP1 protein expression was noticeably higher in gastric cancer tissues than in adjacent normal tissues (Fig. 1a and Supplementary Fig. 1f). Importantly, high G3BP1 expression was positively associated with tumour progression, lymph node metastasis, advanced TNM stage and vascular invasion in gastric cancer patients (Supplementary Table 1). Furthermore, patients with high G3BP1 expression (*n* = 245) displayed a poorer survival rate than patients with low G3BP1 expression (*n* = 210) (*P* < 0.001; Fig. 1b).

We retrospectively separated the advanced gastric cancer (stages II–IV, *n* = 383) patients into two groups according to the type of postoperative treatment: a surgery following ACT group (*n* = 132) and a surgery-alone group (*n* = 251). We then evaluated the correlation of G3BP1 expression with the survival rate. Among the ACT patients, those with high expression of G3BP1 showed a significantly lower survival rate than those with low G3BP1 expression (*P* = 0.008) (Fig. 1c). However, G3BP1 expression was not associated with the survival rate of patients within the surgery-alone group (*P* = 0.157) (Fig. 1d). To validate our observation that G3BP1 is positively correlated with gastric cancer chemotherapy, we performed an integrated microarray dataset-based analysis of the GSE14210, GSE15459, GSE22377, GSE29272, GSE51105 and GSE62254 datasets. The results showed that high G3BP1 expression was significantly associated with poor OS and progression-free survival of gastric cancer patients in the cohort who received postoperative chemotherapy, but not in the surgery-alone group (Fig. 1e, f). Taken together, these observations suggest that high G3BP1 expression can predict or influence the response of gastric cancer patients to chemotherapy.

### G3BP1 enhances gastric cancer chemoresistance both in cells and in vivo

**Fig. 2:**
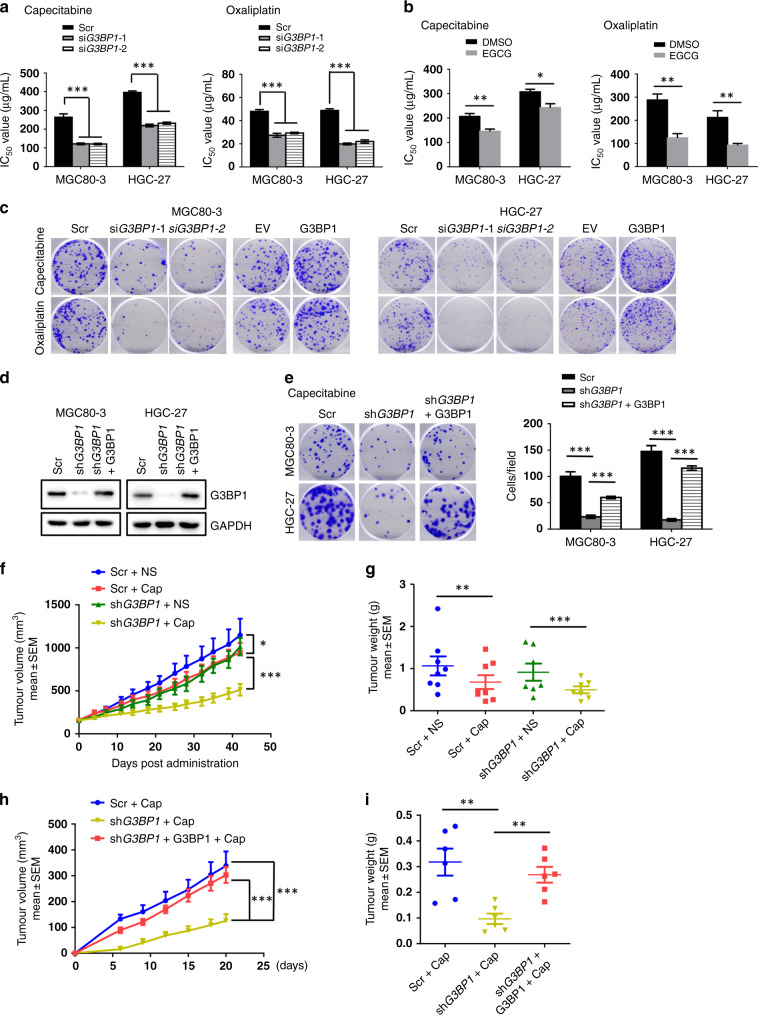
G3BP1 enhances gastric cancer resistance to chemo-drugs in vitro and in vivo. **a** MGC80-3, HGC-27 cells were transfected with scramble or si*G3BP1* and treated with capecitabine or oxaliplatin at gradient concentrations for 24 hours, and the IC_50_ values were examined by CCK-8 assay. Data were shown as mean ± SD of three independent experiments. ***P < 0.001. **b** The IC_50_ values of capecitabine and oxaliplatin combined with or without EGCG treatment (25 μM, 48 h) were examined by CCK-8 assay. Data were shown as mean ± SD of three independent experiments. *P < 0.05, **P < 0.01. **c** Representative images of colony formation assay of MGC80-3 and HGC-27 cells transfected with the indicated siRNAs or plasmids under capecitabine (16 μg/mL) or oxaliplatin (2 μg/mL) treatment. Scale bar: 5 mm. **d** Immunoblotting analysis (IB) of G3BP1 protein expression in MGC80-3 and HGC-27 cells stably transfected with the indicated plasmids. **e** Cells generated in **d** were assessed for colony formation ability under capecitabine (16 μg/mL, 2 weeks) treatment. Scale bar: 5 mm. The statistic results of colony formation assay were shown on the right panel. Data were shown as mean ± SD of three independent experiments. ***P < 0.001. **f**, **g** The stable MGC80-3 cell lines were generated after transfection with scramble or sh*G3BP1* plasmids and then 1 × 10^7^ cells were injected subcutaneously into 6-week-old nude mice. Starting 2 weeks later, mice were randomly allocated into the chemotherapy group (injection with 200 mg/kg capecitabine intraperitoneally, three times per week) or control group (normal saline). After 6 weeks post injection, tumour growth curves were constructed (**f**), and dissected tumours were collected and weighed (**g**). Data represented the mean ± SEM. Two-way ANOVA analysis was used to compare the difference between groups. *P < 0.05, **P < 0.01 and ***P < 0.001. **h**, **i** Different groups of MGC80-3 cells (1 × 10^7^ cells) were injected subcutaneously into the flanks of 6-week-old NOD-SCID mice. At 6 days after the injection, three groups of NOD-SCID mice were injected with capecitabine intraperitoneally (100 mg/kg, two times per week). Tumour size was monitored (**h**), and dissected tumour were weighed (**i**). Data represented the mean ± SEM. Two-way ANOVA analysis was used to compare the difference between groups. **P < 0.01 and ***P < 0.001. Scr scramble, DMSO dimethylsulfoxide, EGCG epigallocatechin gallate, EV empty vector, NS normal saline, cap capecitabine.

Fluoropyrimidine-based chemotherapy (5-fluorouracil, capecitabine or S-1) and platinum-based drugs (cisplatin or oxaliplatin) are the first-in-class chemo-drugs for advanced gastric cancer treatment.^22^ They function by disrupting normal RNA processing and function^23^ or by activating the DNA damage response and the induction of mitochondrial apoptosis.^24^ Therefore, to further evaluate whether G3BP1 governs gastric cancer chemoresistance, we examined G3BP1 function in gastric cancer cells following capecitabine and oxaliplatin treatments. After depleting *G3BP1* in gastric cancer cells (Supplementary Fig. 2a), we examined the IC_50_ values of these two drugs in suppressing gastric cancer cell growth and observed that depletion of *G3BP1* markedly decreases the IC_50_ values of both capecitabine and oxaliplatin in gastric cancer cells (Fig. 2a and Supplementary Fig. 2b). Consistent with this finding, pharmacological inhibition of G3BP1 with the specific inhibitor EGCG greatly reduced the IC_50_ of capecitabine and oxaliplatin (Fig. 2b). Moreover, upon treatment of capecitabine or oxaliplatin, ectopically expressed G3BP1 greatly increased, whereas depletion of *G3BP1* decreased the colony-forming ability of gastric cancer cells compared to that of parental cells (Fig. 2c and Supplementary Fig. 2c, d). More importantly, re-introduction of G3BP1 could largely rescue the *G3BP1* depletion-induced repression of colony-forming capability (Fig. 2d, e).

Next, to validate these findings, we conducted xenograft tumorigenesis experiments in nude mice. We found that *G3BP1* depletion and capecitabine treatment had synergistic effects in markedly reducing subcutaneous tumour growth compared to that in the control group, indicating that sh*G3BP1* gastric cancer cells were likely more sensitive to chemotherapy following capecitabine treatment (Fig. 2f, g and Supplementary Fig. 2e, f). Importantly, re-introduction of G3BP1 in gastric cancer cells could efficiently rescue *G3BP1*-deficiency-reduced tumorigenesis (Fig. 2h, i and Supplementary Fig. 3), indicating that G3BP1 plays an important role in the chemoresistance of gastric cancer cells both in cells and in vivo.

### G3BP1 inhibits gastric cancer cell apoptosis and pro-apoptotic gene expression

**Fig. 3:**
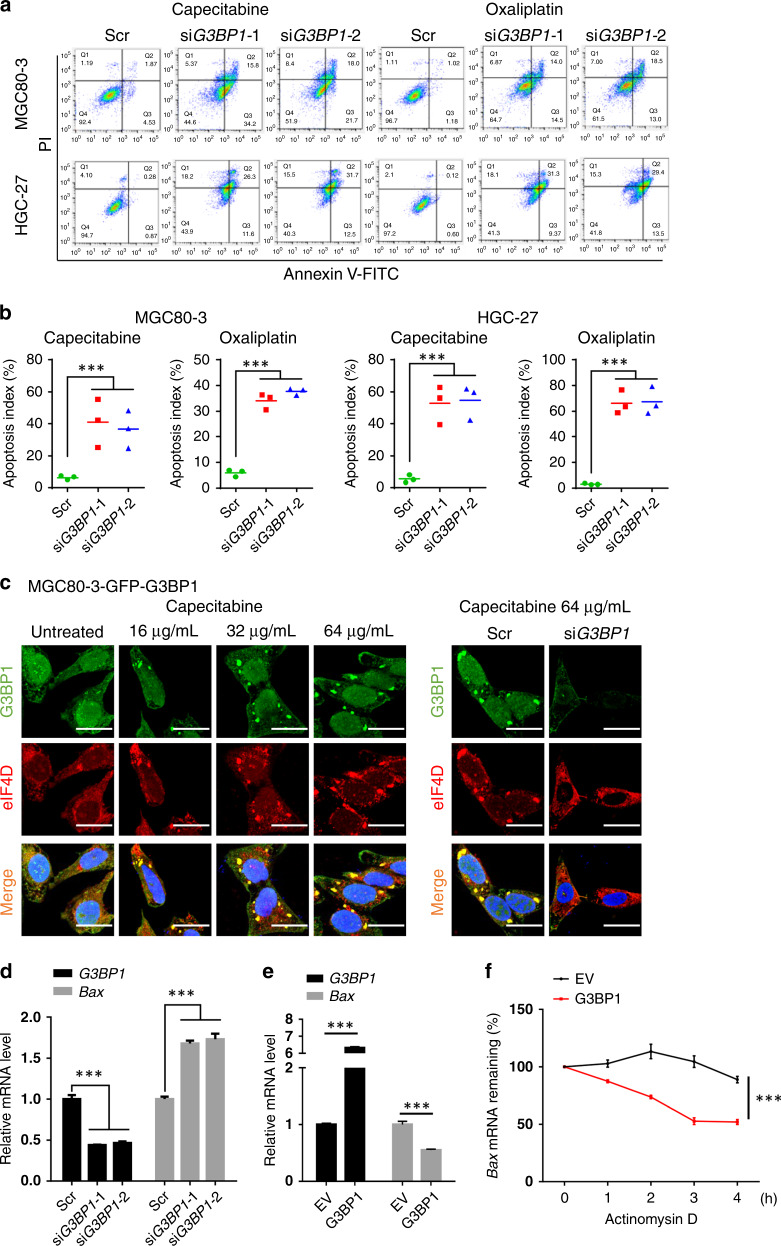
G3BP1 inhibits apoptosis and Bax expression by regulating stress granules formation. **a** MGC80-3 and HGC-27 cells transfected with scramble or si*G3BP1* were treated with capecitabine (64 μg/mL) or oxaliplatin (8 μg/mL) for 12 h and then stained with annexin V-FITC and propidium iodide (PI) for FACS analysis. **b** The quantification of annexin V and PI-positive cells related to **a**. Data were shown as mean ± SD of three independent experiments. ***P < 0.001. **c** Representative images of immunofluorescence assay. Left: MGC80-3 were stably transfected with pCDH-GFP-G3BP1 plasmids, and then was treated with capecitabine (64 μg/mL, 24 h). The cells were fixed and stained by using anti-G3BP1 (1:50) or anti-eIF4D (1:100) antibodies. Right: MGC80-3-GFP-G3BP1 cells were transfected with scramble or si*G3BP1*, exposed to capecitabine (64 μg/mL, 24 h) and stained by using anti-G3BP1 (1:50) or anti-eIF4D (1:100) antibodies. Scale bar: 25 μm. **d** MGC80-3 cells transfected with scramble or si*G3BP1* were treated with capecitabine (64 μg/mL) for 24 h and then were harvested for RT-PCR analysis. Data were shown as mean ± SD of three independent experiments. ***P < 0.001. **e** MGC80-3 cells transfected with pcDNA3.1 (EV) or pcDNA3-HA-G3BP1 and then were treated with capecitabine (64 μg/mL) for 24 h. The cells were harvested for RT-PCR analysis. Data were shown as mean ± SD of three independent experiments. ***P < 0.001. **f** MGC80-3 cells treated as **e** were incubated with actinomysin D (5 μg/mL) and then were harvested for RT-PCR analysis. Data were shown as mean ± SD of three independent experiments. ***P < 0.001. Scr scramble, EV empty vector.

Chemotherapy resistance arises due to the dysregulation of multiple processes, such as drug transport, drug metabolism, DNA repair and apoptosis protection.^24–27^ Of note, the dysregulation of pro-apoptotic/anti-apoptotic factors or pathways is attracting increasing research attention. Consistent with the finding that *G3BP1* knockdown increased gastric cancer sensitivity to chemotherapy, depletion of *G3BP1* substantially increased the apoptosis level in gastric cancer cell lines, including MGC80-3, HGC-27, SGC-7901 and BGC-823, as indicated by the Annexin V-FITC and PI labelling-based flow cytometry analysis (Fig. 3a, b and Supplementary Fig. 4a, b). However, the cell cycle of GC cells was mildly influenced by *G3BP1* depletion (Supplementary Fig. 4c). In addition, another apoptosis marker, the TUNEL (terminal deoxynucleotidyl transferase dUTP nick-end labelling) score, of MGC80-3 xenograft tumours was significantly increased in the *G3BP1*-depleted group compared to the control group upon capecitabine treatment, while the proliferation index (Ki-67 score) was largely decreased in the sh*G3BP1* group relative to the control group (Supplementary Fig. 4d–f). We next examined the expression of apoptotic-related factors and found that pro-apoptotic markers (cleaved PARP, cleaved caspase-9, cleaved caspase-3 and Bax) were markedly elevated in *G3BP1*-compromised gastric cancer cells (Supplementary Fig. 5a, b).

As it is well known, G3BP1 functions as an effector of SGs assembly and regulates mRNA turnover during cellular stress.^15^ We thus examined SGs formation under chemo-drug treatment. The results showed that capecitabine induced SGs formation in a dose-dependent manner (Fig. 3c). However, SGs formation was significantly reduced under the condition of *G3BP1* depletion (Fig. 3c). Subsequently, we detected the mRNA levels of *Bax* in MGC80-3 cells and found that G3BP1 could significantly reduce the mRNA abundance and mRNA half-life of *Bax* in gastric cancer cells (Fig. 3d, f). Collectively, these results suggest that in response to anti-cancer drug treatment, G3BP1 confers gastric cancer chemotherapeutic resistance through inhibiting cellular apoptosis.

### G3BP1 is co-expressed with YWHAZ to facilitate anti-apoptosis and drug resistance

**Fig. 4:**
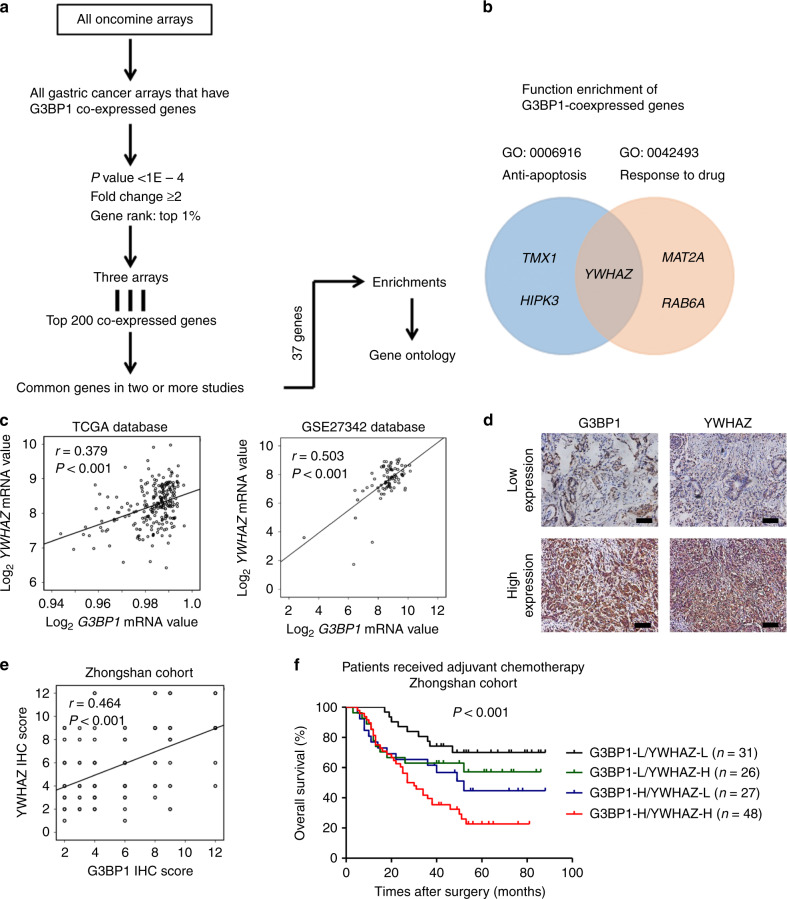
G3BP1 is co-expressed with YWHAZ in gastric cancer. **a** Methodological workflow of G3BP1 co-expression analysis. Top 200 co-expressed genes met the criteria from three gastric cancer arrays were selected. Thirty-seven genes presenting in at least two arrays were finally included and put to functional enrichment analyses. **b** Functional enrichment of G3BP1 co-expressed genes in anti-apoptosis or response to drug GO pathways. **c** Correlation of *G3BP1* and *YWHAZ* mRNA expression in gastric cancer from TCGA database (*n* = 232) and GEO database (GSE27342, *n* = 80). **d** Representative images of IHC results showing G3BP1 and YWHAZ expression in consecutive gastric tumour sections. Scale bar: 100 µm. **e** The correlation of G3BP1 and YWHAZ IHC scores from Zhongshan cohort patients (*n* = 455). **f** Kaplan–Meier analysis for overall survival of advanced gastric cancer patients receiving adjuvant chemotherapy according to the expression of G3BP1 and YWHAZ in Zhongshan cohort (*n* = 132). L low expression, H high expression.

To explore the mechanism by which G3BP1 confers anti-apoptosis activity and chemoresistance in gastric cancer cells, we conducted gene co-expression analyses by using the Oncomine database^28^ (Fig. 4a). Thirty-seven genes were identified as co-expressed with G3BP1 in more than two studies from three different arrays: Chen Gastric (Stanford), Forster Gastric (GSE22377) and Kim Gastric (GSE14208) (Supplementary Table 2). After functional enrichment analysis of these 37 genes by using the web-based Database for Annotation, Visualisation and Integrated Discovery (http://david.abcc.ncifcrf.gov), we found that three co-expressed genes, *TMX3*, *HIPK3* and *YWHAZ*, were annotated with the Gene Ontology term “anti-apoptosis”, whereas three co-expressed genes, *MAT2A*, *RAB6A* and *YWHAZ*, were annotated as “response to drugs” (Fig. 4b and Supplementary Table 3). The associations of these candidate genes with the survival of gastric cancer patients were analysed by using an online database. We found that only *YWHAZ* displayed a significant correlation with the survival of gastric cancer patients who received postoperative chemotherapy (Supplementary Fig. 6a–e).

Then, we investigated the correlation between YWHAZ expression and G3BP1 expression in human gastric cancer samples. We found that *G3BP1* mRNA levels were positively correlated with *YWHAZ* mRNA levels in gastric cancer patients by data mining the TCGA database (*n* = 233, *r* = 0.379, *P* < 0.001) and the GEO database (*n* = 80, *r* = 0.503, *P* < 0.001) (Fig. 4c). Further analysis of Zhongshan cohort patients by IHC staining demonstrated that the protein expression of G3BP1 and that of YWHAZ were also positively correlated in human gastric cancer (*n* = 455, *r* = 0.464, *P* < 0.001) (Fig. 4d, e). Consistent with a previous report,^29^ YWHAZ was highly expressed in gastric cancer tissues compared with adjacent normal tissues from Zhongshan cohort patients (*n* = 455, *P* < 0.001) (Supplementary Fig. 7a, b). Importantly, high YWHAZ expression indicated a poor outcome for gastric cancer patients (Supplementary Fig. 7c). Moreover, we found that the survival rate of G3BP1^high^YWHAZ^high^ patients (*n* = 48) was significantly lower than that of patients bearing either G3BP1^high^YWHAZ^low^ (*n* = 27) or G3BP1^low^YWHAZ^high^ (*n* = 26) gastric cancer after ACT. As expectedly, the patients with low expression of both G3BP1 and YWHAZ (*n* = 31) displayed the best survival rate among the four groups (Fig. 4f). These results suggest that the biomarker considering the expression of both G3BP1 and YWHAZ has superior power in the prediction of the prognosis of gastric cancer patients receiving ACT.

### G3BP1 interacts with YWHAZ to sequester the apoptosis regulator Bax into the cytoplasm

**Fig. 5:**
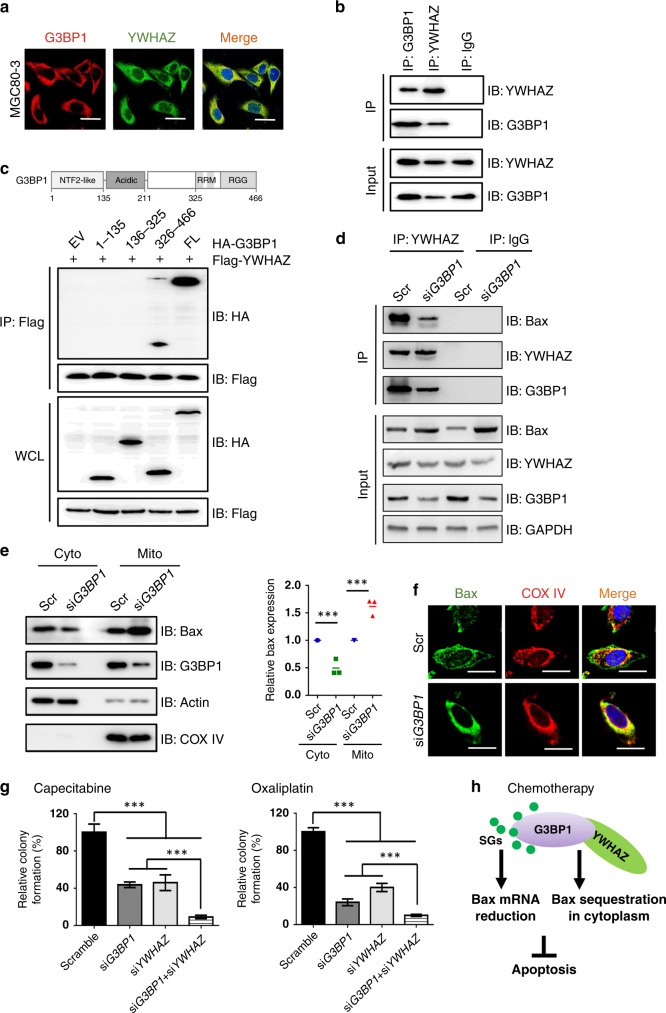
G3BP1 interacts with YWHAZ to sequester the apoptosis regulator Bax in the cytoplasm. **a** Representative images of the co-localisation of G3BP1 and YWHAZ in MGC80-3 cells. Cells were incubated with anti-G3BP1 (1:50) and anti-YWHAZ (1:50) antibodies. Scale bar: 25 µm. **b** Co-immunoprecipitation (co-IP) experiments in MGC80-3 cells were performed using anti-G3BP1 or anti-YWHAZ antibodies. IgG was used as a negative control. **c** IB analysis of WCLs and immunoprecipitates (IPs) derived from 293T cells transfected with Flag-YWHAZ together with the indicated constructs of HA-G3BP1. **d** Co-IP experiments in MGC80-3 cells transfected with scramble or si*G3BP1* were performed using anti-YWHAZ antibody or anti-rabbit IgG as control. **e** Left: IB analysis of cell cytoplasm and mitochondria fractions separated from MGC80-3 cells transfected with scramble or si*G3BP1*. Right: The quantification of relative Bax protein expression. Data were shown as mean ± SD of three independent experiments. ***P < 0.001. **f** Representative images of Bax localisation in MGC80-3 cells transfected with scramble or si*G3BP1* under the treatment of capecitabine (64 μg/mL) for 24 h. Cells were fixed and stained by using anti-Bax (1:50) or anti-COX IV (1:100) antibodies. COX IV staining was indicated as the localisation of mitochondria. Scale bar: 25 µm. **g** The statistical results of colony formation assay of MGC80-3 cells transfected with the indicated siRNAs under capecitabine (16 μg/mL, 2 weeks) or oxaliplatin (2 μg/mL, 2 weeks) treatment. Data were shown as mean ± SD of three independent experiments. ***P < 0.001. **h** The proposed model for G3BP1 in regulating tumour apoptosis and chemoresistance in gastric cancer. Scr scramble, IP immunoprecipitation, IB immunoblotting, WCL whole-cell lysate, cyto cytoplasm, mito mitochondria, SGs stress granules.

To address the molecular mechanisms by which the co-expression and co-operative function of G3BP1 and YWHAZ act in gastric cancer, we initially performed immunofluorescence assay to detect the intracellular localisation of G3BP1 and YWHAZ in gastric cancer cells. The results showed that G3BP1 and YWHAZ were co-localised in the cytoplasm of the examined cancer cells (Fig. 5a and Supplementary Fig. 8a). Consistently, the reciprocal interaction of endogenous G3BP1 and YWHAZ was proved in MGC80-3 cells (Fig. 5b). To more precisely identify the binding regions of G3BP1 with YWHAZ, we generated several HA-tagged G3BP1 fragments and found that the RRM and RGG domains (326–466 aa) of G3BP1 were preferred binding sites for YWHAZ (Fig. 5c).

Nomura et al.^30^ and Tsuruta et al.^31^ observed that upon stress stimuli, JNK (c-Jun N-terminal kinase) could phosphorylate YWHAZ at Ser-184 and reduce its affinity for Bax, resulting in Bax dissociation from YWHAZ and redistribution to mitochondria. This observation indicated that YWHAZ could negatively regulate apoptosis by binding and sequestering Bax in the cytoplasm. Therefore, we speculated that G3BP1 co-expression and binding with YWHAZ could modulate the interaction between YWHAZ and Bax in gastric cancer cells. Interestingly, our immunoprecipitation result showed that the interaction between YWHAZ and Bax was significantly reduced in *G3BP1*-depleted MGC80-3 cells (Fig. 5d). Furthermore, the expression of Bax in the mitochondria, where Bax exhibits the pro-apoptosis effect, was markedly increased in MGC80-3 cells upon *G3BP1* depletion (Fig. 5e), which might be ascribed to the reduced interaction and sequestration of Bax by YWHAZ. This finding was supported by the confocal imaging data (Fig. 5f). Although G3BP1 formed a complex with YWHAZ and Bax (Fig. 5a and Supplementary Fig. 8a–c), the knockdown of *YWHAZ* or *Bax* had little effect on SGs formation in GC cells (Supplementary Fig. 8d).

To further verify the effects of G3BP1 and YWHAZ on chemoresistance, we constructed a chemotherapeutic drug-resistant GC cell line by culturing MGC80-3 cells with continuous exposure to capecitabine or oxaliplatin for 3 months (Supplementary Fig. 9a). Interestingly, we found that both G3BP1 and YWHAZ were upregulated in capecitabine/oxaliplatin-resistant MGC80-3 cells (Supplementary Fig. 9b). Moreover, knockdown of *G3BP1* or *YWHAZ* displayed a larger impact on IC_50_ values in chemo-resistant MGC80-3 cells than in parental cells (Supplementary Fig. 9c), further supporting a role of G3BP1/YWHAZ in chemoresistance.

We next investigated whether the combined knockdown of *G3BP1* and *YWHAZ* could exert a synergetic effect on the inhibition of GC proliferation. Colony formation assays revealed that *YWHAZ* depletion enhanced the colony formation ability of *G3BP1* deficiency-suppressed MGC80-3 cells upon capecitabine or oxaliplatin treatment (Fig. 5g and Supplementary Fig. 9d). This finding was consistent with our previous finding that among gastric cancer patients receiving chemotherapy, those with G3BP1^low^YWHAZ^low^ expression displayed the best survival rate, whereas the G3BP1^high^YWHAZ^high^ patients had the lowest.

## DISCUSSION

Chemotherapy resistance is a complex and multifactorial phenomenon that is associated with a wide range of molecular mechanisms. Chemotherapy resistance can be classified into several types: (i) pre-target resistance, related to processes that precede the binding of chemotherapeutic drugs to its target; (ii) on-target resistance, related to the molecular damage provoked by chemotherapeutic drugs; (iii) post-target resistance, related to the lethal signalling pathways triggered by molecular lesions and (iv) off-target resistance, related to influence on molecular circuitries that are not intimately associated with drugs-elicited signals.^12,24^ Programmed cell death, commonly referred to as apoptosis, is proposed to be a common pathway that mediates the killing effects of chemotherapeutic drugs.^32^ The apoptosis pathway acts as a key determinant of cancer chemoresistance by enhancing the expression of anti-apoptotic factors and suppressing the expression of pro-apoptotic proteins,^33^ which includes Bcl-2 family members, TP53 and mismatch-repair or DNA-repair system proteins.^34,35^ SGs formation is a kind of self-protection method for cells to avoid chemotherapy-induced apoptosis. Cancer cells, exposed to the conditions of hypoxia, nutrient starvation or chemotherapy-induced high reactive oxygen species, are expected to promote eukaryotic initiator factor 2A phosphorylation-triggered SGs assembly.^36^ In addition, it has been established that SGs act as storage sites for non-translating mRNAs separated from disassembled polysomes;^13^ thus, G3BP1, an essential component of SGs, might also be involved in the regulation of apoptosis and the response to chemotherapy in gastric cancer, as investigated in this study.

Here, we found that G3BP1 had prognostic and predictive value regarding ACT benefits in patients with gastric cancer. Further mechanistic exploration revealed that G3BP1 could inhibit pro-apoptotic *Bax* mRNA expression and suppress apoptosis in gastric cancer cells treated with chemotherapy. G3BP1 functions as an SGs assembly factor, regulating mRNA turnover and determining mRNA fates during cellular stress.^37^ In degrading the mRNA of targeted genes, G3BP1 exerts its physiological roles as a cytosine and adenine (CA) dinucleotide-specific endoribonuclease and binds specific RNA sequences with its RRM domain.^15,38^ It was reported that G3BP1 degraded *GAS5*, *IGF-II* and other target mRNAs in mouse embryonic fibroblast (MEF) cells and the mRNA half-life of these mRNAs was prolonged in *G3bp1*^−/−^ MEFs.^15^ Similarly, in our study, we observed that G3BP1 could regulate the mRNA abundance and mRNA half-life of *Bax* in gastric cancer cells, revealing the pathological roles of G3BP1 in response to chemo-drug treatment and chemoresistance.

In addition, we found through a gene co-expression analysis that G3BP1 could be co-expressed with YWHAZ in gastric cancer. Gene co-expression analysis is used for functional classification, gene–disease prediction and regulatory gene identification. Based on the microarray or RNA-sequencing data, co-expression network analysis can be used to construct networks of genes with tendencies to be co-activated in the same biological processes; these networks can then be used to identify genes with regulatory roles in disease and their correlations with one another.^39^ Here, we found that in human gastric cancer, YWHAZ was the essential gene co-expressed with G3BP1 and that it was involved in both anti-apoptosis and drug resistance. Further investigation showed that YWHAZ was positively correlated with G3BP1 expression in gastric cancer patients and that high expression of both G3BP1 and YWHAZ was associated with the worst prognosis in gastric cancer patients who received ACT. Our mechanistic exploration showed that G3BP1 co-localised with YWHAZ in the cytoplasm and physiologically interacted with YWHAZ in gastric cancer cells. In turn, G3BP1 enhanced the interaction between YWHAZ and Bax and caused the sequestration of Bax into the cytoplasm, resulting in reduced Bax retention in mitochondria, where Bax exerts its pro-apoptotic effects. Meanwhile, knockdown of *YWHAZ* or *Bax* had little effect on SGs formation in gastric cancer cells, which indicates that G3BP1/YWHAZ/Bax pathway exerts anti-apoptosis and chemoresistance roles in gastric cancer via an SG-independent mechanism (Fig. 5h).

Taken together, in this study, G3BP1 was revealed to govern gastric cancer apoptosis and to play a pivotal role in gastric cancer chemoresistance through mediating SGs formation or via an SG-independent mechanism: the G3BP1/YWHAZ/Bax axis. Our findings not only provide an improved prognostic marker for gastric cancer chemoresistance but also suggest a potential strategy to target G3BP1 for sensitising gastric cancer to chemotherapy.

## Supplementary information

Supplementary materials

## Data Availability

The data that support the findings of this study are available from the corresponding author upon reasonable request.
