# Supplementary material for: G3BP1 interacts with YWHAZ to regulate chemoresistance and predict adjuvant chemotherapy benefit in gastric cancer
Source: Br J Cancer. 2020 Sep 29;124(2):425–36. doi: 10.1038/s41416-020-01067-1 (PMC7852868; doi:10.1038/s41416-020-01067-1)
Supplement: Supplementary file 1 — Supplementary materials [file 41416_2020_1067_MOESM1_ESM.pdf]

Supplementary Fig. 1

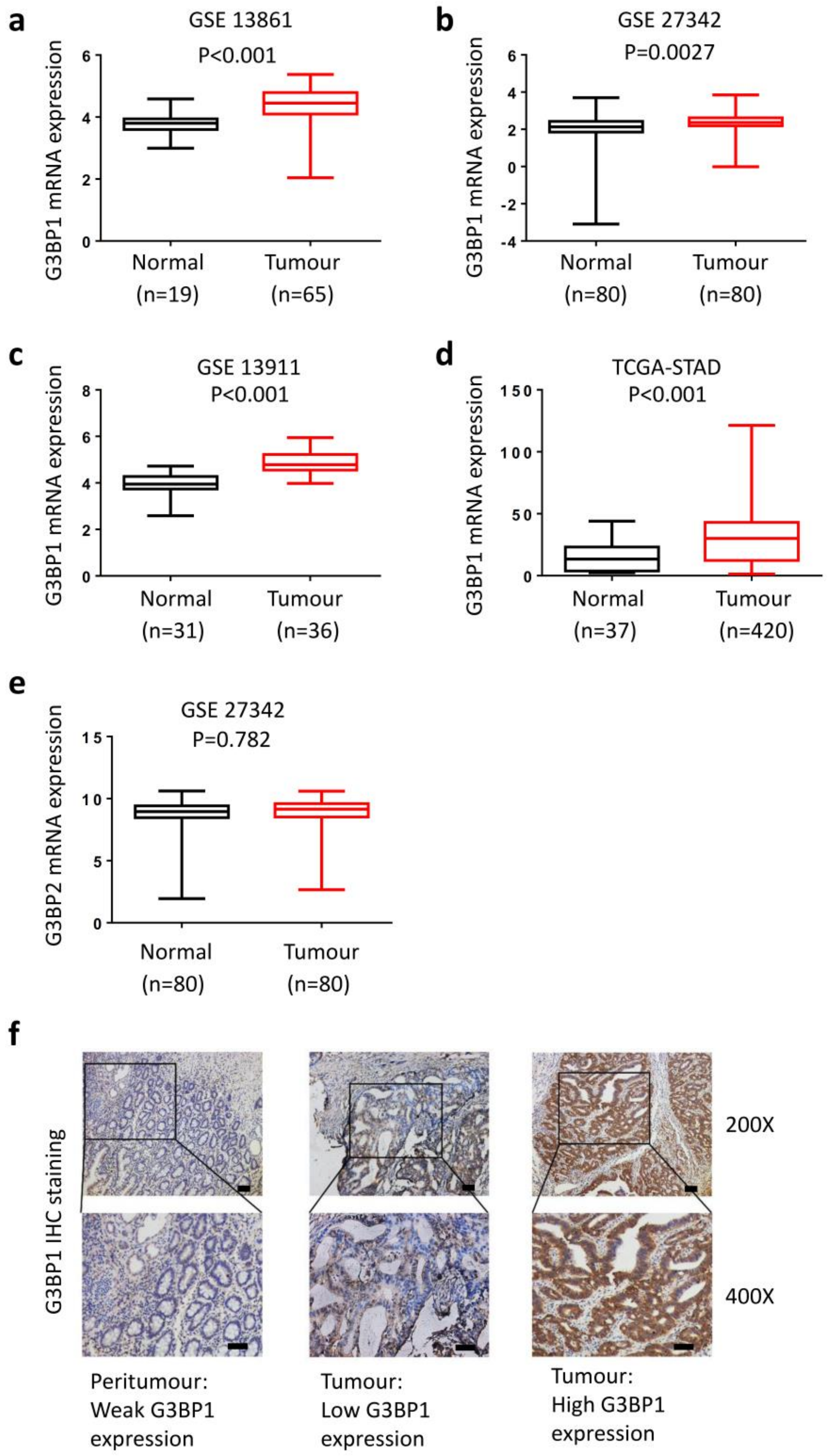

Supplementary Fig. 1. The expression of G3BP1 but not G3BP2 was up-regulated in gastric cancer

(a-d) The mRNA expression level of *G3BP1* in three datasets from GEO database

(GSE13861, GSE27342, GSE13911) and TCGA database. **(e)** The mRNA expression level of *G3BP2* in GEO database (GSE27342). **(f)** Representative images of G3BP1 IHC staining in peritumour and tumour tissues and its corresponding regional magnification images. Low and high expression of G3BP1 in tumour sections was determined by ROC analysis. Scale bar: 100 μm. The box plot showed the full range of variation.

Supplementary Fig. 2

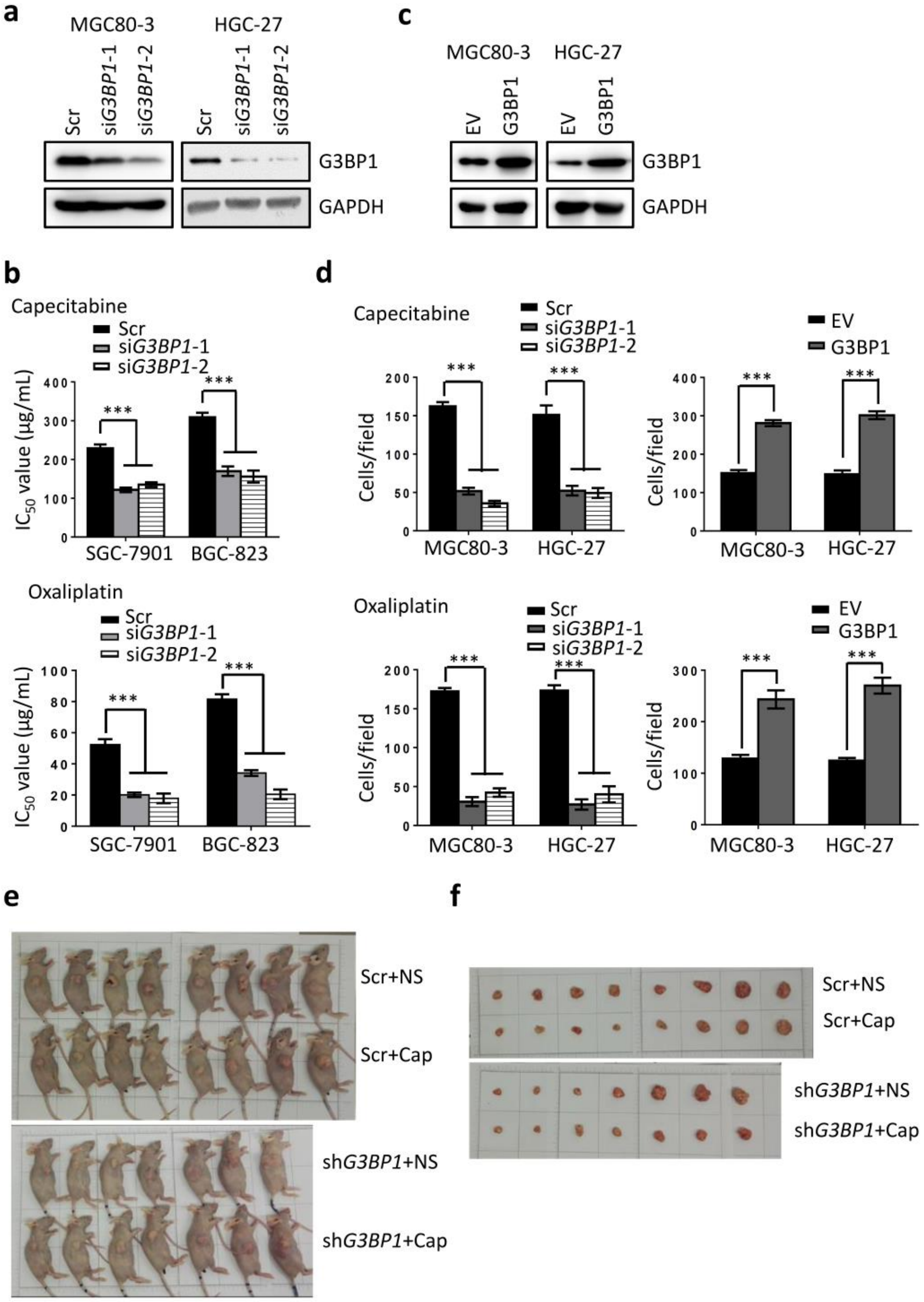

Supplementary Fig. 2. G3BP1 enhanced gastric cancer resistance to chemo-drugs *in vitro* and *in vivo*

(a) Immunoblotting (IB) analysis of whole cell lysates (WCLs) derived from MGC80-3 and HGC-27 cells transfected with Scramble or si*G3BP1*. **(b)** SGC-7901 and BGC-

823 cells were transfected with scramble or si*G3BP1* and treated with capecitabine or oxaliplatin at gradient concentrations for 24 hours and the IC_{50} values were examined by CCK-8 assay. The data were shown as mean ± SD of three independent experiments. ***P < 0.001. **(c)** IB analysis of WCLs derived from MGC80-3 and HGC-27 cells transfected with pcDNA3.1 or pcDNA3-G3BP1. **(d)** The statistic results of colony formation assay refer to Fig. 2c. The data were shown as mean ± SD of three independent experiments. ***P < 0.001. **(e-f)** Different groups of MGC80-3 cells (1×10^7 cells) were injected subcutaneously into the flanks of 6-week-old male Balb/c nude mice. When tumour volume reached approximately 100 mm^3, each Balb/c nude mouse was randomly allocated into the chemotherapy group (injection with 200 mg/kg capecitabine intraperitoneally, 3 times per week) or control group (injection with normal saline). At 6 weeks post injection, the Balb/c nude mice were respectively sacrificed and tumour tissues were harvested. Images of four groups of mice were shown in **(e)** and the collected tumors were shown in **(f)**. Scr: scramble; EV: empty vector; NS: normal saline; Cap: capecitabine.

Supplementary Fig. 3

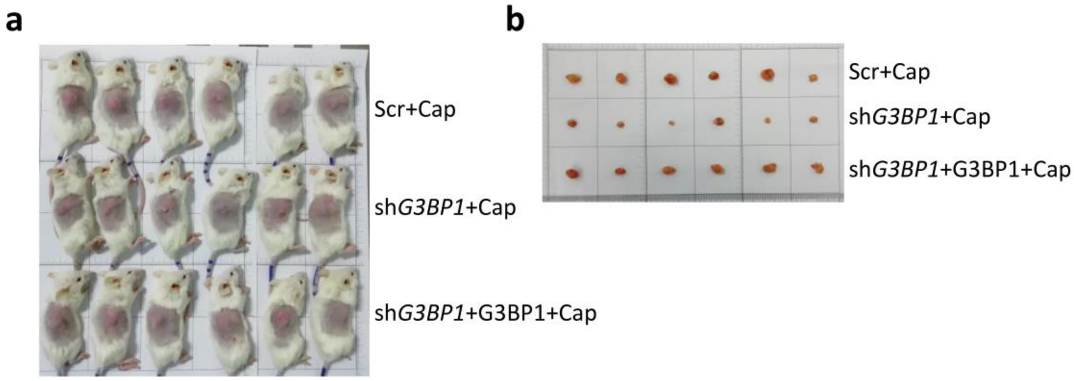

Supplementary Fig. 3. Ectopic G3BP1 could reverse the reduced tumorigenesis of sh*G3BP1* gastric cancer cells

(a-b) Different groups of MGC80-3 cells (1×10^7 cells) were injected subcutaneously into the flanks of 6-week-old male NOD-SCID mice. At 6 days after the injection, the NOD-SCID mice were injected with capecitabine intraperitoneally (100 mg/kg, 2 times per week). 3weeks later, the mice were sacrificed and tumor tissues were harvested. Images of three groups of NOD-SCID mice were shown in (a) and the tumors were shown in (b). Scr: scramble; Cap: capecitabine.

Supplementary Fig. 4

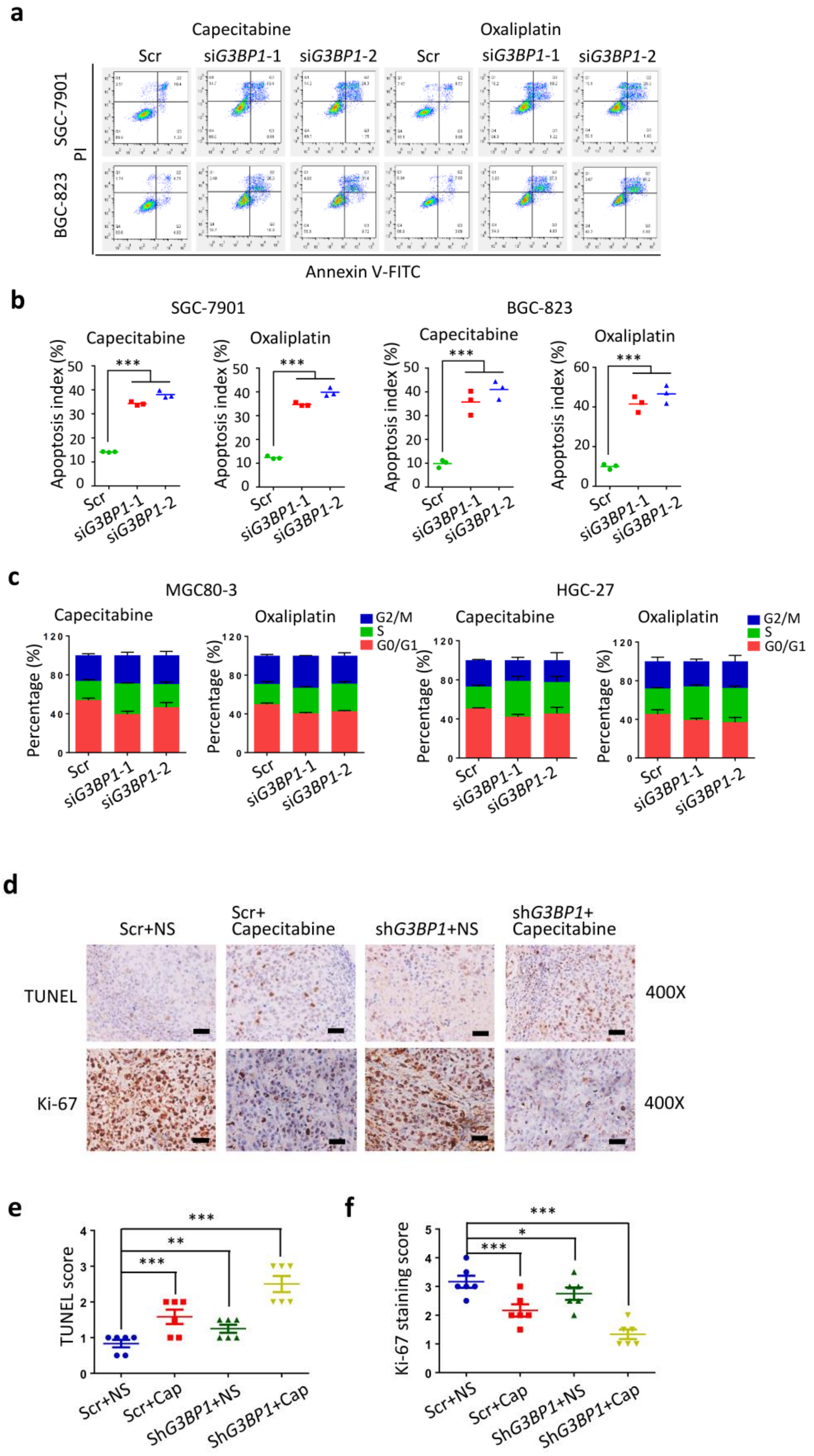

Supplementary Fig. 4. The apoptosis level but not the cell cycle was significantly changed in gastric cancer cells when G3BP1 was knocked down

(a-b) SGC-7901 and BGC-823 cells transfected with scramble or si*G3BP1* were treated with capecitabine (64 μg/mL) or oxaliplatin (8 μg/mL) for 12 hours and then stained with annexin V-FITC and propidium iodide (PI). Representative images were shown in **(a)** and quantification charts were shown in **(b)**. Data were shown as mean ± SD of three independent experiments. ***P < 0.001. **(c)** MGC80-3 and HGC-27 cells transfected with scramble or si*G3BP1* were treated with capecitabine (64 μg/mL) or oxaliplatin (8 μg/mL) for 12 hours and then stained with propidium iodide (PI) for cell cycle analysis. Data were shown as mean ± SD of three independent experiments. **(d)** Representative images of TUNEL and Ki-67 IHC staining in subcutaneous tumour sections. Scale bar: 100 μm. **(e-f)** Statistical graphs of TUNEL or Ki-67 IHC staining score. Data were shown as mean ± SD. *P < 0.05, **P < 0.01, ***P < 0.001. Scr: scramble; NS: normal saline; Cap: capecitabine.

Supplementary Fig. 5

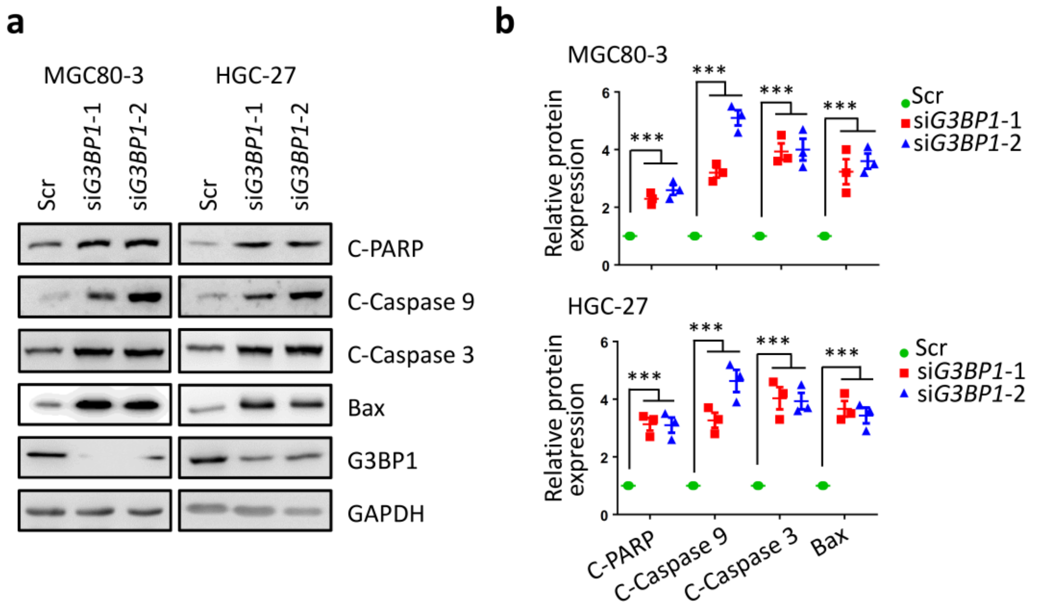

Supplementary Fig. 5. Pro-apoptotic markers were elevated in gastric cancer cells when *G3BP1* was knocked down

(a) IB analysis of WCLs derived from MGC80-3 and HGC-27 cells transfected with the indicated siRNAs by using anti-Cleaved PARP, anti-Cleaved Caspase-9, anti-Cleaved Caspase-3 or anti-Bax antibody. **(b)** The quantification results refer to **(a)**. Data were shown as mean ± SD of three independent experiments. ***P < 0.001. Scr: scramble.

Supplementary Fig. 6

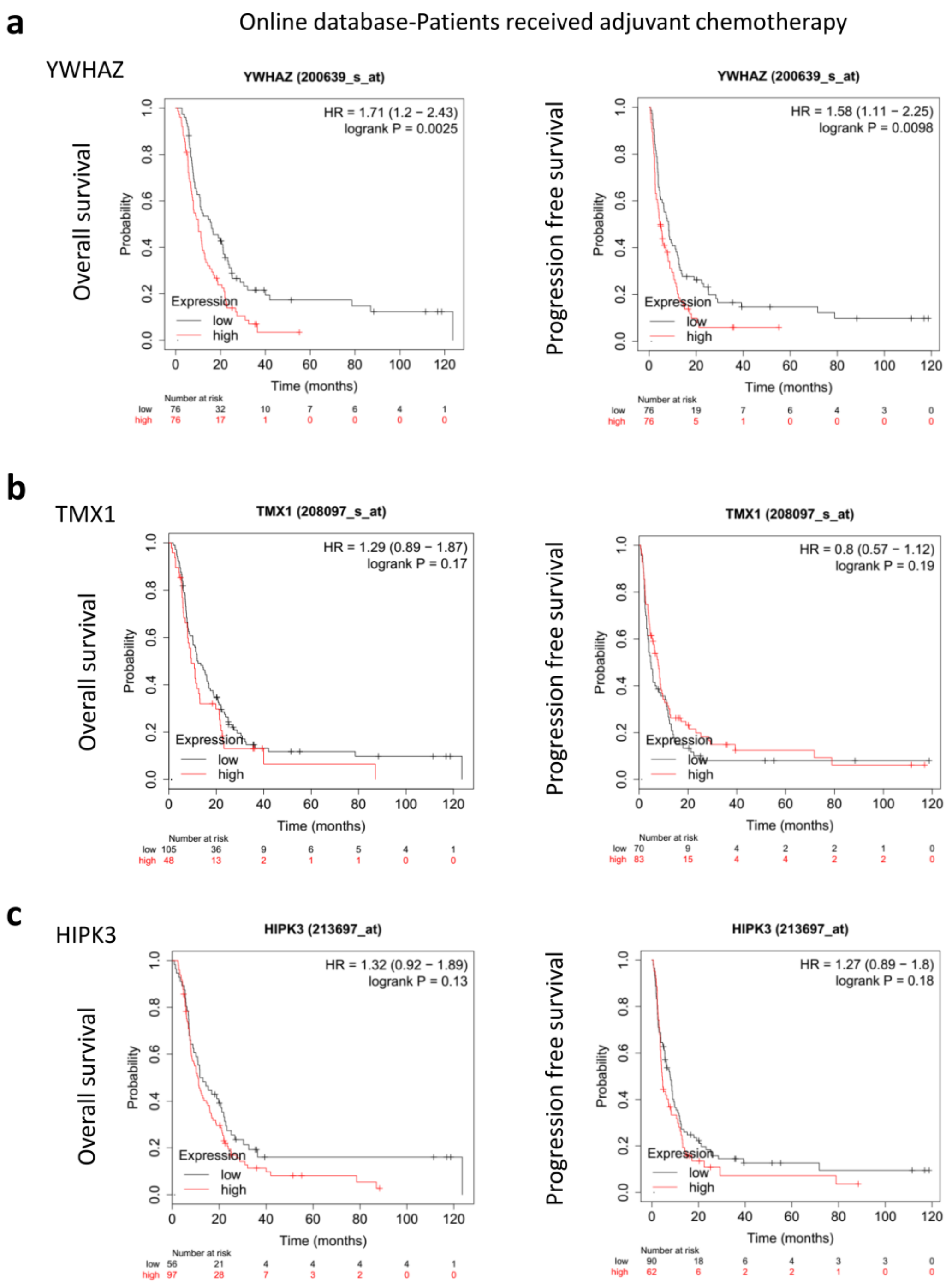

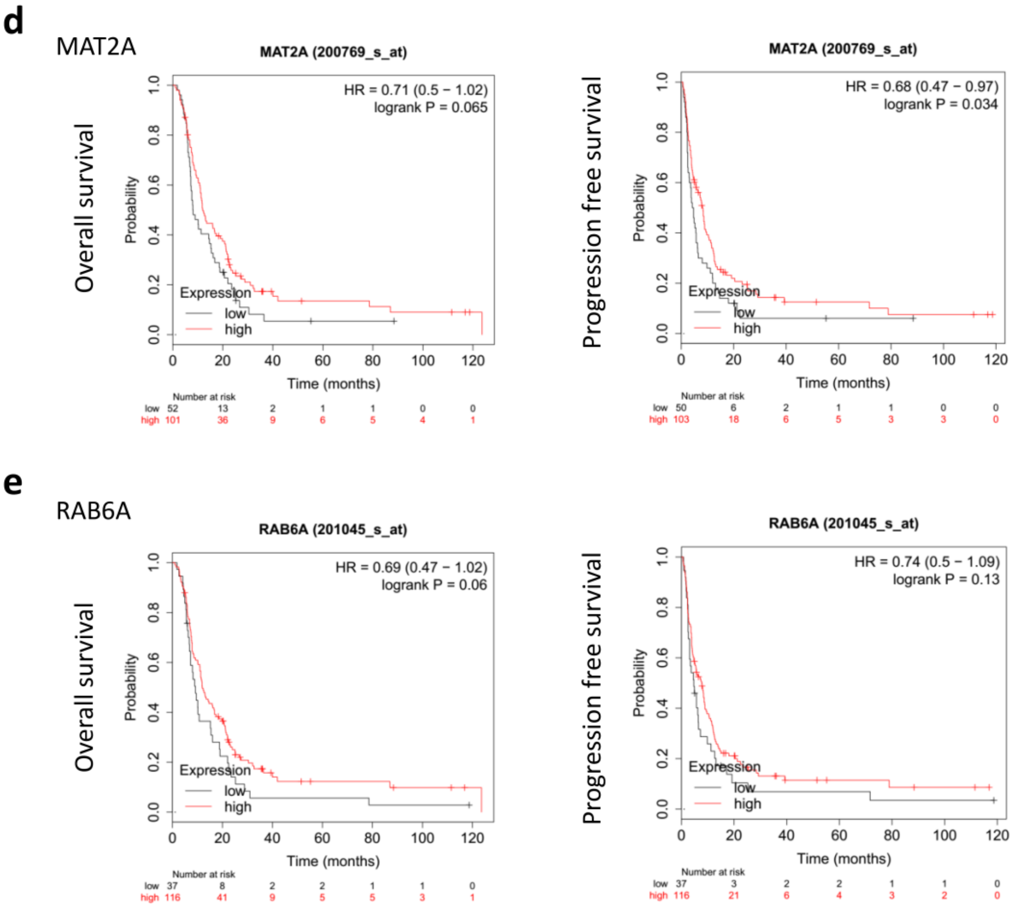

Supplementary Fig. 6. Online database analysis showed that YWHAZ was correlated with poor outcome of gastric cancer patients receiving adjuvant chemotherapy

(a-e) The association of *YWHAZ* **(a)**, *TMX1* **(b)**, *HIPK3* **(c)**, *MAT2A* **(d)** and *RAB6A* **(e)** mRNA expression with overall survival or progression-free survival of gastric cancer patients receiving adjuvant chemotherapy by analyzing the GEO datasets (http://www.kmplot.com/analysis/index.php?p=service&cancer=gastric).

Supplementary Fig. 7

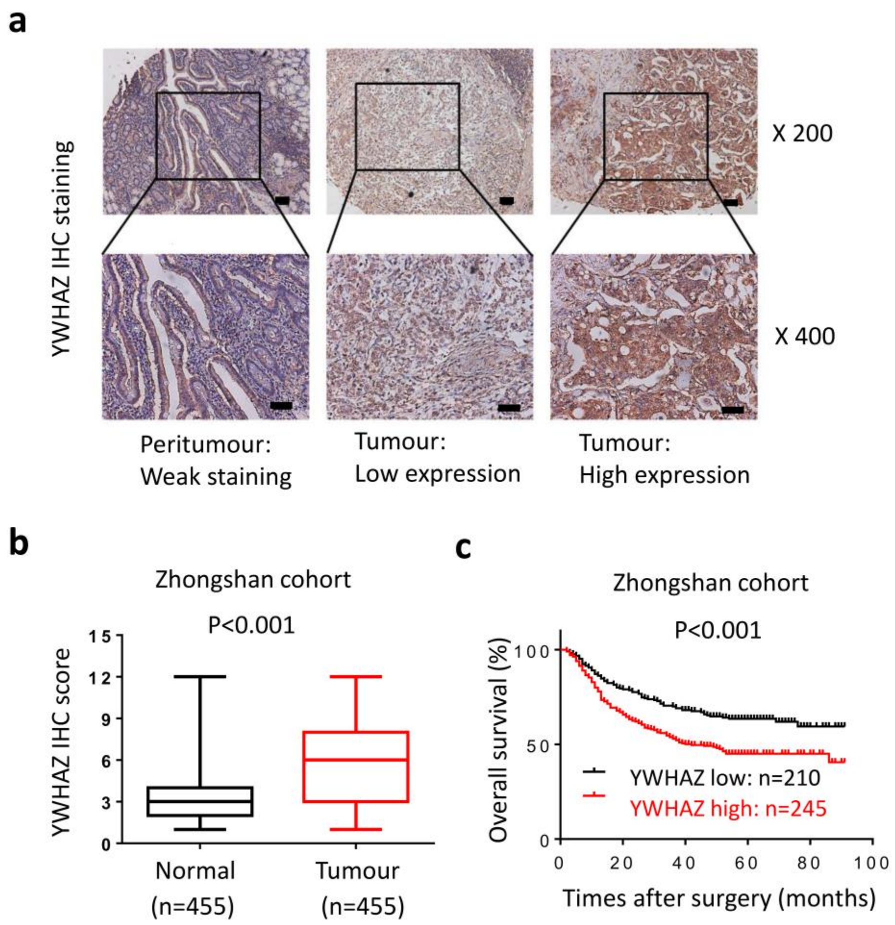

Supplementary Fig. 7. YWHAZ was overexpressed and associated with worse prognosis in gastric cancer patients from Zhongshan cohort

(a) Representative images of YWHAZ IHC staining in peritumour and tumour tissues and its corresponding regional magnification images. Low and high expression of YWHAZ in tumour sections was determined by ROC analysis. Scale bar: 100 μm. **(b)** YWHAZ IHC scores in normal and tumour tissues from Zhongshan cohort (n=455). The box plot showed the full range of variation (error bars: min and max) with the line representing median. **(c)** Kaplan-Meier analysis for overall survival of gastric cancer patients according to YWHAZ expression (n=455).

Supplementary Fig. 8

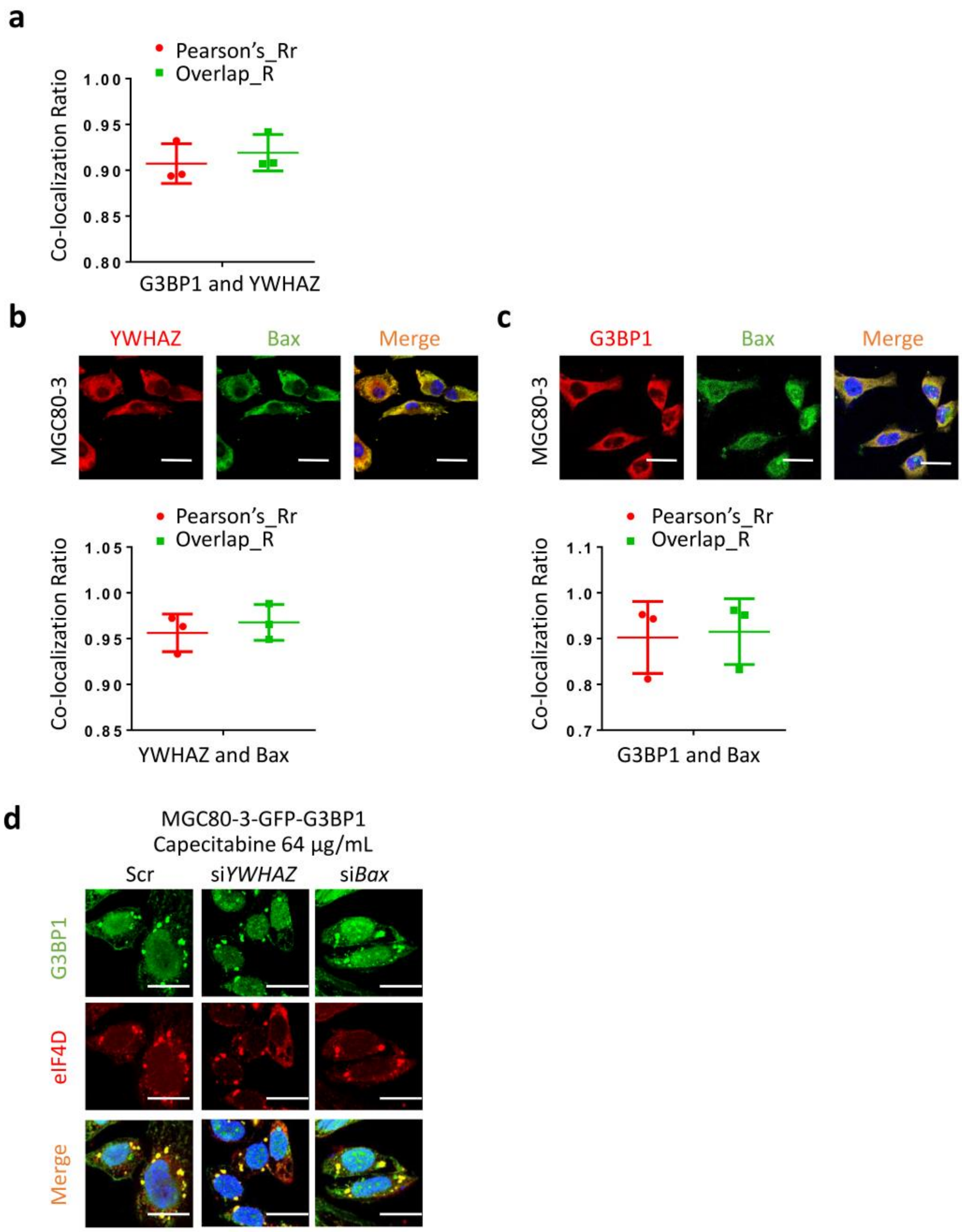

Supplementary Fig. 8. Bax co-localized with YWHAZ and G3BP1

(a) The ratio quantification of co-localization in Fig. 5a. Both Pearson's correlation and Overlap coefficient were calculated by ImageJ software. **(b)** Representative images of co-localization of YWHAZ and Bax in MGC80-3 cells (upper panel) and the quantification was shown in lower panel. Scale bar: 25 μm. **(c)** Representative images

of co-localization of G3BP1 and Bax in MGC80-3 cells (upper panel) and the quantification was shown in lower panel. Scale bar: 25 μm. In a-c, data were shown as mean ± SD of three independent experiments. **(d)** Representative images of immunofluorescence. MGC80-3-GFP-G3BP1 cells were transfected with scramble, si*YWHAZ* or si*Bax*, and then were treated with capecitabine (64 μg/mL, 24 hours). The cells were fixed and stained by using anti-G3BP1 (1:50) and anti-eIF4D (1:100) antibodies. Scale bar: 25 μm.

Supplementary Fig. 9

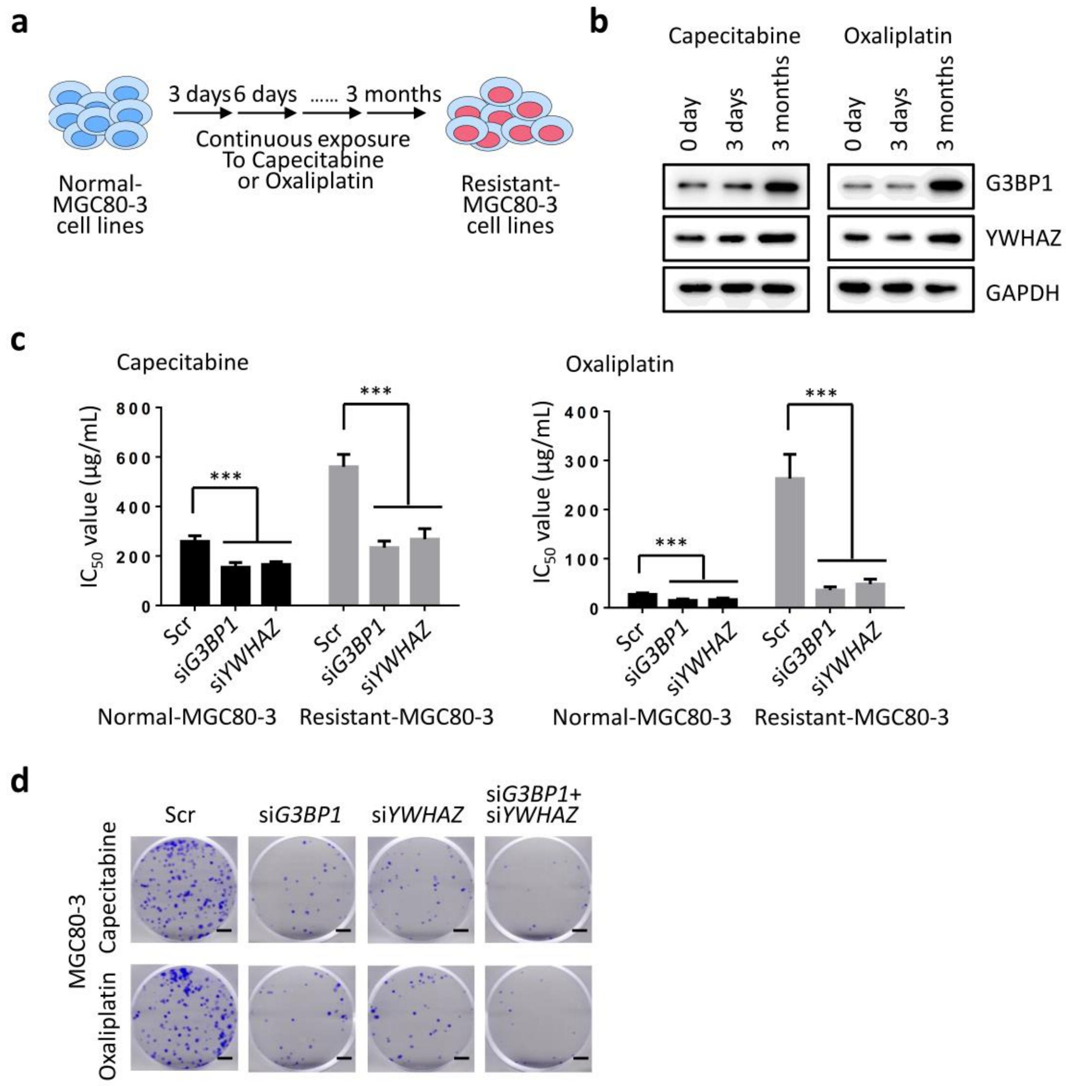

Supplementary Fig. 9. G3BP1 and YWHAZ were upregulated and displayed larger impact on IC_{50} values in drug-resistant cells

(a) Schematic diagram showed the construction process of drug-resistant MGC80-3 cell lines. MGC80-3 cells were exposed to capecitabine or oxaliplatin with gradient increased concentration for 3 months. **(b)** Representative gel images of G3BP1 or YWHAZ protein expression in Normal MGC80-3 cells or cells exposed to chemo-drugs for 3 days or 3 months. **(c)** Normal-MGC80-3 or resistant-MGC80-3 cells were treated with capecitabine or oxaliplatin for 48 hours and the IC_{50} values were examined

by CCK-8 assay. Data were shown as mean ± SD of three independent experiments. ***$P < 0.001$. **(d)** The representative images of colony formation assay in Fig. 5g. Scale Bar: 5 mm. Scr: scramble.

Supplementary Table 1. Relation between intratumoral G3BP1 expression and clinical characteristics of gastric cancer in Zhongshan cohort

Factor	Patients	G3BP1 expression		
	No. (%)	Low	High	P-value
**All patients**	455 (100)	210	245	
**Age (years)**				0.928
≤65	177 (38.9)	89	88	
>65	278 (61.1)	141	137	
**Gender**				0.665
Female	147 (32.3)	70	77	
Male	308 (67.7)	140	168	
**Localization**				**0.048**
Proximal	75 (16.5)	31	44	
Middle	97 (21.3)	36	61	
Distal	283 (62.2)	143	140	
**Differentiation**				0.684
Well + Moderately	86 (18.9)	38	48	
Poorly	369 (81.1)	172	197	
**Lauren classification**				**0.015**
Intestinal type	278 (61.1)	114	164	
Diffuse type	151 (33.2)	84	67	
Mixed type	26 (5.7)	12	14	
**T classification**				**<0.001**
T1	67 (14.7)	54	13	
T2	39 (8.6)	21	18	
T3	141 (31.0)	57	84	
T4	208 (45.7)	78	130	
**N classification**				**<0.001**
N0	123 (27.0)	73	50	
N1	78 (17.1)	44	34	
N2	90 (19.8)	43	47	
N3	164 (36.1)	50	114	
**Distant metastasis**				0.259
No	442 (97.1)	206	236	
Yes	13 (2.9)	4	9	
**TNM stage**				**<0.001**
I	72 (15.8)	57	15	
II	112 (24.6)	53	59	
III	258 (56.7)	96	162	
IV	13 (2.9)	4	9	
**Vascular invasion**				**<0.001**
No	322 (70.8)	171	151	
Yes	133 (29.2)	39	94	

Abbreviation: TNM=tumour node metastasis. P-value < 0.05 marked in bold font shows statistical significant.

Supplementary Table 2. G3BP1-coexpressed genes from online database

No.	gene symbol	Correlation	No.	gene symbol	Correlation
1	CAPRIN1	0.779	20	ADAM10	0.649
2	TOMM22	0.764	21	ZFR	0.643
3	SNRNP40	0.764	22	ATP6AP2	0.643
4	SEPT11	0.721	23	HNRNPU	0.623
5	ZNF207	0.691	24	SCARB2	0.598
6	HNRNPH1	0.672	25	GNS	0.598
7	EIF2S3	0.658	26	RAB6A	0.590
8	YWHAZ	0.649	27	G3BP2	0.590
9	TMX1	0.649	28	RNFT1	0.562
10	TMOD3	0.649	29	CDV3	0.509
11	SCAMP1	0.649	30	TBL1XR1	0.486
12	MAT2A	0.649	31	DCUN1D1	0.486
13	LIN7C	0.649	32	TMEM30A	0.465
14	HIPK3	0.649	33	SYPL1	0.465
15	EXOC5	0.649	34	ZMYND11	0.445
16	DDX3X	0.649	35	CUL4B	0.431
17	CD164	0.649	36	C5orf24	0.431
18	ATF2	0.649	37	TMED2	0.422
19	AGPS	0.649			

Supplementary Table 3. Functional enrichment of G3BP1-coexpressed genes

Term	Count	%	P-value	Fold	FDR
GO:0016192-vesicle-mediated transport	7	18.9	0.001	5.5	1.615
GO:0006887-exocytosis	4	10.8	0.002	15.7	2.509
GO:0008104-protein localization	8	21.6	0.002	4.1	2.891
GO:0015031-protein transport	7	18.9	0.005	4.1	6.453
GO:0045184-establishment of protein localization	7	18.9	0.005	4.1	6.738
GO:0032940-secretion by cell	4	10.8	0.01	8.7	12.362
GO:0046903-secretion	4	10.8	0.026	6.1	29.971
GO:0006916-anti-apoptosis	3	8.1	0.032	10.3	35.706
GO:0042493-response to drug	3	8.1	0.042	8.8	44.523
GO:0000375-RNA splicing, via transesterification reactions	3	8.1	0.042	8.8	44.523
GO:0000377-RNA splicing, via transesterification reactions with bulged adenosine as nucleophile	3	8.1	0.042	8.8	44.523
GO:0046907-intracellular transport	5	13.5	0.05	3.4	50.295
GO:0006626-protein targeting to mitochondrion	2	5.4	0.07	26.5	63.086
GO:0070585-protein localization in mitochondrion	2	5.4	0.07	26.5	63.086
GO:0048193-Golgi vesicle transport	3	8.1	0.072	6.6	63.723
GO:0000398-nuclear mRNA splicing, via spliceosome	3	8.1	0.078	6.3	66.856
GO:0009057-macromolecule catabolic process	5	13.5	0.083	2.9	69.465
GO:0007162-negative regulation of cell adhesion	2	5.4	0.088	20.9	71.657
GO:0002274-myeloid leukocyte activation	2	5.4	0.094	19.6	74.047
GO:0045321-leukocyte activation	3	8.1	0.094	5.6	74.154

Fold, fold enhancement; FDR, false discovery rate.
